# Production of a recombinant swollenin from *Trichoderma harzianum* in *Escherichia coli* and its potential synergistic role in biomass degradation

**DOI:** 10.1186/s12934-017-0697-6

**Published:** 2017-05-16

**Authors:** Clelton A. Santos, Jaire A. Ferreira-Filho, Anthonia O’Donovan, Vijai K. Gupta, Maria G. Tuohy, Anete P. Souza

**Affiliations:** 1 Molecular Glycobiotechnology Group, Department of Biochemistry, National University of Ireland Galway, Galway, Ireland; 2 Center for Molecular Biology and Genetic Engineering, University of Campinas, Campinas, SP Brazil; 3 Technology Centre for Biorefining and Bioenergy, Orbsen Building, National University of Ireland, Galway, Ireland; 4 Department of Plant Biology, Biology Institute, University of Campinas, Campinas, SP Brazil; 5 Department of Chemistry and Biotechnology, ERA Chair of Green Chemistry, School of Science, Tallinn University of Technology, Tallinn, Estonia

**Keywords:** *Trichoderma harzianum*, Swollenin, Expansin, Overexpression, Biomass degradation

## Abstract

**Background:**

Fungal swollenins (SWOs) constitute a class of accessory proteins that are homologous to canonical plant expansins. Expansins and expansin-related proteins are well known for acting in the deagglomeration of cellulose structure by loosening macrofibrils. Consequently, SWOs can increase the accessibility and efficiency of the other enzymes involved in the saccharification of cellulosic substrates. Thus, SWOs are promising targets for improving the hydrolysis of plant biomass and for use as an additive to enhance the efficiency of an enzyme cocktail designed for the production of biofuels.

**Results:**

Here, we report the initial characterization of an SWO from *Trichoderma harzianum* (ThSwo) that was successfully produced using *Escherichia coli* as a host. Initially, transcriptome and secretome data were used to compare *swo* gene expression and the amount of secreted ThSwo. The results from structural modeling and phylogenetic analysis of the ThSwo protein showed that ThSwo does preserve some structural features of the plant expansins and family-45 glycosyl hydrolase enzymes, but it evolutionarily diverges from both of these protein classes. Recombinant ThSwo was purified at a high yield and with high purity and showed secondary folding similar to that of a native fungal SWO. Bioactivity assays revealed that the purified recombinant ThSwo created a rough and amorphous surface on Avicel and displayed a high synergistic effect with a commercial xylanase from *T. viride*, enhancing its hydrolytic performance up to 147 ± 7%.

**Conclusions:**

Many aspects of the structure and mechanism of action of fungal SWOs remain unknown. In the present study, we produced a recombinant, active SWO from *T. harzianum* using a prokaryotic host and confirmed its potential synergistic role in biomass degradation. Our work paves the way for further studies evaluating the structure and function of this protein, especially regarding its use in biotechnology.

**Electronic supplementary material:**

The online version of this article (doi:10.1186/s12934-017-0697-6) contains supplementary material, which is available to authorized users.

## Background

The enzymatic degradation of lignocellulosic substrates is a cooperative process that relies on the simultaneous synergistic actions of different specialized proteins [1–4]. During the last few decades, several studies have focused on the search for and characterization of enzymes involved in biomass degradation [5–9]. However, an increasing number of recent studies have attempted to elucidate the role of accessory and non-catalytic proteins in catabolic pathways and to identify how these proteins could be used to enhance the enzymatic hydrolysis of cellulose [10, 11].

Expansins and expansin-related proteins, which are non-enzymatic proteins, are well known for acting in plant biomass degradation [10]. The expansin superfamily comprises a group of proteins found in plants, amoebas, nematodes, fungi and bacteria that can act in the deagglomeration of cellulose and other cell wall polysaccharides by loosening macrofibrils [12–15]. Consequently, these proteins increase the accessibility and efficiency of the others enzymes involved in the overall process of saccharification.

The best-known expansin-related protein in fungi is called swollenin (SWO) [13, 16]. Fungal SWO is an interesting example of an expansin-like protein because it differs considerably in size from the classical plant expansin: the plant expansins are typically no larger than 250 amino acid residues, whereas the SWO in fungi is twice this size. This size difference further supports the hypothesis that the evolution of bacterial and fungal expansin-related proteins occurred via horizontal gene transfer and independent domain fusion events [17]. In addition, SWO shows a unique mode of action that differs from that of plant expansins but that is similar to the action of both endoglucanases and cellobiohydrolases, which do not fit into any canonical cellulose class [16].

Although there are questions regarding whether SWO should be considered an expansin [18], SWO has recently been detected in the secretome of important cellulolytic fungi species, including *Penicillium* sp., *Aspergillus* sp. and *Trichoderma* sp., and has been shown to improve enzymatic cocktails designed for biomass degradation [19–24]. Based on these findings, new approaches for producing and purifying SWOs are attractive to the biofuels industry.

In the present study, we developed a novel protocol for the expression and purification of a recombinant, active SWO from *T. harzianum* (ThSwo) using *E*. *coli* as a host. *T*. *harzianum*, one of the best-known saprophytic fungi, produces a wide range of cellulose-active proteins that can be exploited for biotechnological purposes. The recombinant ThSwo was purified at a high yields and with high purity, allowing for its characterization using different biochemical and biophysical techniques. Phylogenetic analysis and protein structural modeling were also performed. Available transcriptome and secretome data on *T. harzianum* growth under biomass degradation conditions were used to quantify both relative ThSwo gene expression and actual protein production. Finally, the synergistic effect of purified recombinant ThSwo on the enzymatic hydrolysis of cellulose was investigated.

## Results and discussion

### Structural modeling and phylogenetic analysis of ThSwo

**Fig. 1:**
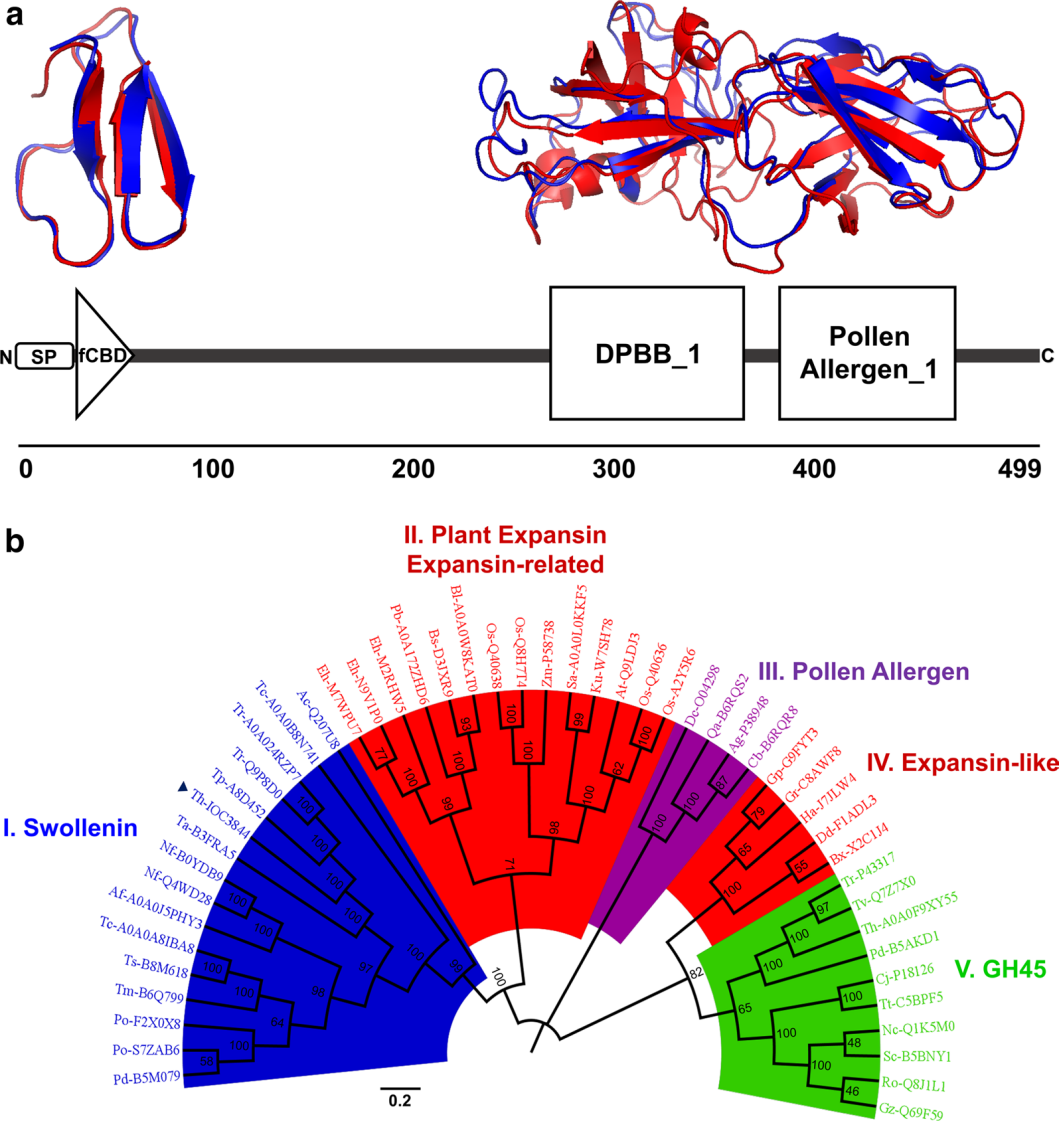
ThSwo protein architecture, modeling and phylogenetic relationships with expansin-related proteins. **a** *Bottom* scheme of the ThSwo domain organization; *top* ThSwo protein structure prediction. The best-ranked N- and C-terminal ThSwo model (*blue*) was superimposed on the C-terminal domain of cellobiohydrolase I from *T. reesei* (PDB code: 2CBH) and the structure of maize beta-expansin (PDB code: 2HCZ), respectively, highlighted in *red* using PYMOL software. **b** Phylogenetic divergence between ThSwo and representative plant expansins, expansin-related proteins from bacteria, fungi, amoebozoa and nematodes, pollen allergen proteins and family-45 glycosyl hydrolases (GH45). The bootstrap values are shown at each node. The complete list of sequences and species name abbreviations used to construct the tree are given in Additional file 1: Table S1. The ThSwo sequence (Th-IOC3844) is identified with an* arrow*

Initially, we used bioinformatic tools to identify and model the ThSwo topology and domain organization (Fig. 1a). ThSwo, similar to other fungal SWOs, is a modular protein [13] composed of an N-terminal region encompassing a signal peptide (amino acid residues 1–25) and a fungal-type cellulose-binding domain (fCBD; amino acid residues 24–57) spaced by a linker (residues 58–272) of the C-terminal region, which in turn encloses two other domains, the double-psi beta-barrel fold (DPBB; residues 273–372) followed by a pollen allergen_1 domain (residues 387–475), as predicted using the SMART server [25] (Fig. 1a, bottom). The ThSwo C-terminal region is intriguing because, although the DPBB domain is structurally related to family-45 glycosyl hydrolases (GH45), plant extracts containing proteins of group-I pollen allergens are active in loosening cell walls [18, 26]. Subsequently, since no three-dimensional SWO structures are available in the Protein Data Bank (PDB; http://www.rcsb.org/) to date, structural modeling approaches using the I-TASSER server were also performed to provide more information about the structural features of ThSwo (Fig. 1a, top). The models generated with the full-length protein sequence showed low confidence scores (C-scores). The C-score is used by I-TASSER to estimate the quality of predicted models [27]; for this reason, the N-terminal fCBD and the C-terminal (DPPD and pollen allergen_1) domains were used separately in an attempt to generate high-quality models. The generated models individually converged with high C-score values and were used for structural protein alignment using protein structures available in the PDB (Fig. 1a, top). While the predicted ThSwo fCBD model showed a remarkable fit with the C-terminal domain of cellobiohydrolase I from *Trichoderma reesei* (PDB code: 2CBH; [28]), the ThSwo C-terminal model was closely related to the structure of EXPB1 (PDB code: 2HCZ), a beta-expansin and group-1 pollen allergen from maize that resembles the folding of GH45 endoglucanases [29]. The modeling findings are strengthened by reported results showing that SWO from *T. reesei* has unusual enzymatic kinetics, sharing similarities with the action of both endoglucanases and cellobiohydrolases [16]. However, further studies to resolve the three-dimensional structure of ThSwo should be conducted to accurately identify the relationship between protein structure and function.

The phylogenetic analysis between ThSwo and expansin-related proteins from plant, nematode, amoebozoa, fungi and bacteria, which were used to root the tree (Additional file 1: Table S1), revealed marked phylogenetic divergence between SWOs and plant expansins as well as expansin-related, pollen allergen and GH45 proteins (Fig. 1b). ThSwo, identified in the tree as Th-IOC3844, was placed in a group comprising almost exclusively other fungal SWOs, forming a node with sub-branches containing the SWOs of *Trichoderma pseudokoningii* (Tp-A8D452) and *T. reesei* (Tr-Q9P8D0 and Tr-A0A024RZP7). Intriguingly, an amoebozoa swollenin/expansin-like protein from *Acanthamoeba castellanii* (Ac-Q207U8), as annotated in the UniProt database (http://www.uniprot.org/), was the only non-fungal protein present in the SWO group, forming a separate branch with the fungal SWOs.

Regarding the plant expansin/expansin-related protein group, the phylogenetic analysis showed the formation of two subgroups containing proteins from plants, amoebozoa and bacteria. The first expansin subgroup was represented by three proteins from *Entamoeba histolytica* (Eh-M2RHW5, Eh-N9V1P0 and Eh-M7WPU7) and three bacterial proteins (*Paenibacillus bovis* Pb-A0A172ZHD6, *Bacillus subtilis* Bs-D3JXR9 and *Bacillus licheniformis* Bl-A0A0W8KAT0), and the second subgroup contained only plant expansins, with the exception of two bacterial proteins (*Streptomyces acidiscabies* Sa-A0A0L0KKF5 and *Kutzneria* sp. Ku-W7SH78). Notably, *Zea mays* pollen beta-expansin (Zm-P58738), the three-dimensional structure (PDB code: 2HCZ) of which had the best fit with the C-terminal ThSwo model generated by I-TASSER (Fig. 1a, top), was found among the plant expansins used in the phylogenetic analysis. Another expansin-like subgroup containing exclusively nematode expansin-like proteins was also observed; these proteins were distantly related to the plant expansins and more closely related to the GH45 protein family, with 82% bootstrap support.

According to the main hypothesis that explains the evolution of expansin-related proteins in bacteria, fungi and amoebozoa, at least two independent events of expansin gene transfers occurred from plants to microbes, followed by multiple secondary independent vertical/horizontal gene transfer events within amoebozoa, fungi, and bacteria as well as gene domain fusion events, especially in bacteria [17]. Interestingly, the expansin-like proteins in nematodes arose through independent evolution, indicating an evolutionary origin separate from that of plant expansins [17, 30].

Our results show that while the expansin-related proteins in bacteria and amoebozoa have a phylogenetic relationship that is closely intertwined with canonical plant expansins, fungal SWOs constitute a more homogeneous group, probably due to the significant adaptive role that these proteins played in fungal species evolution, although homologous but separate from that of plant expansins.

### ThSwo gene expression and production using *E. coli* as a host

The *swo* gene that encodes ThSwo in the *T. harzianum* IOC-3844 strain was identify by Crucello et al. [31]. Interestingly, *swo* is inserted in a carbohydrate-active enzyme (CAZy)-enriched genomic region that contains three consecutive CAZy genes, including *cbh1* (cellobiohydrolase I) and a xylanase gene (*xyn*) [31]. Moreover, this same structural organization, in terms of gene position within the genome, was also observed in *T. reesei* [32]. Furthermore, in both *T. harzianum* and *T. reesei,* the *cbhI*-*swo*-*xyn* genomic region was co-induced [31, 32]. Thus, gene expression data may be useful in understanding the importance of these genes, especially under conditions of biomass degradation.

**Fig. 2:**
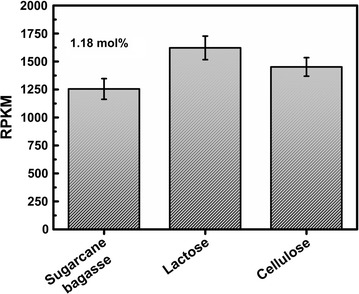
Quantification of ThSwo expression in the transcriptome of *T. harzianum* induced by sugarcane bagasse, lactose and cellulose. ThSwo represents 1.18 mol% of the total secretome of *T. harzianum* cultured on sugarcane bagasse [20]. *RPKM* reads per kilobase per million of mapped reads

The transcriptome profile of *T. harzianum* cultured on delignified sugarcane bagasse (DSB), lactose (LAC) and cellulose (CEL) [33] was used to quantify ThSwo gene expression under these conditions (Fig. 2). The highest values of reads per kilobase per million mapped reads (KPKM) of ThSwo were observed in LAC (1621.7), followed by CEL (1451.7) and DSB (1254.7) (Fig. 2). Although ThSwo expression may be considered low, especially compared to the expression of some β-glucosidases, which can reach approximately 400,000 KPKM [34], ThSwo represents 1.18 mol% of the total secretome of *T. harzianum* cultured on DSB [20]. ThSwo representation in the secretome is especially high compared to the values of 8.32 mol% observed for cellobiohydrolase II, which was the most abundant protein detected in the secretome of *T. harzianum* [20]. This finding confirms that ThSwo is actively used by this fungus under biomass degradation conditions.

**Fig. 3:**
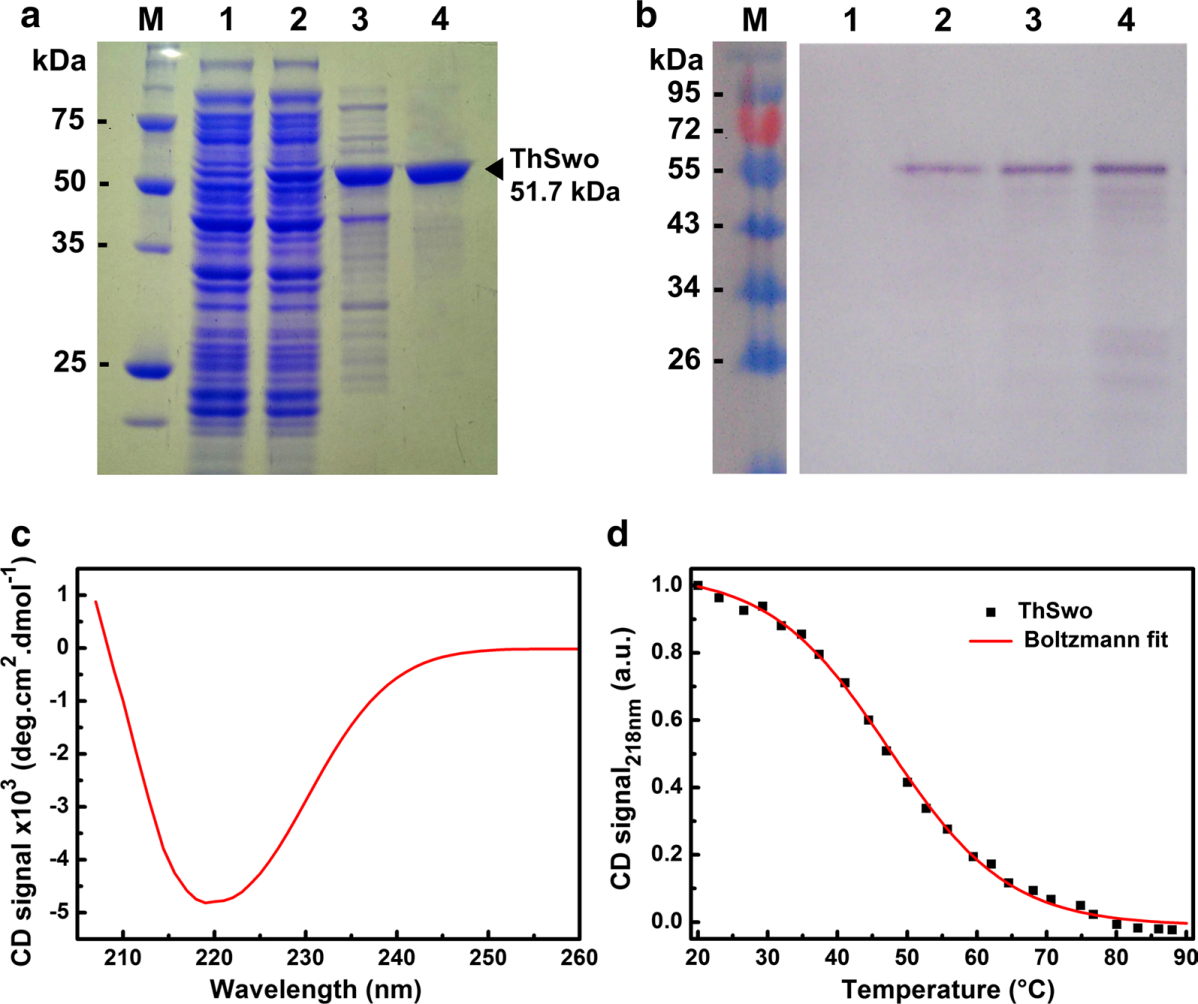
The purification, western blot analysis and secondary folding of recombinant ThSwo. **a** SDS-PAGE (12%) and **b** western blot analysis of ThSwo using a His-probe antibody. *M* molecular mass markers, in both figures **a** and **b**; *lane 1* cell lysate before protein induction; *lane 2* cell lysate after protein induction; *lane 3* protein eluted from nickel affinity chromatography; and *lane 4* proteins eluted from size-exclusion chromatography. **c** Net-smoothed CD spectrum of ThSwo. **d** ThSwo thermal stability followed by CD. The ThSwo thermal-induced unfolding experiments were measured at 218 nm from 20 to 90 °C using approximately 10 µM of purified recombinant ThSwo in buffer A containing 1 mM of TCEP and with a 1 mm pathlength cell. ThSwo had a *Tm* of 48.6 ± 2 °C

Following gene cloning, recombinant ThSwo (494 amino acid residues, 51.7 kDa and a theoretical isoelectric point of 5.5) was successfully expressed using *E. coli* and subsequently purified (Fig. 3a). Different *E. coli* strains including BL21 (DE3), Rosetta, Rosetta-gami 2 (DE3) and C43 were initially used for protein expression assays; however, only the Rosetta-gami 2 (DE3) strain was able to overexpress ThSwo. Rosetta-gami 2 (DE3) is known for enhanced disulfide bond formation in cysteine-rich proteins and enhanced expression of eukaryotic proteins containing codons rarely used in *E. coli*, both of which are characteristics shared by ThSwo. Approximately 10 mg of purified recombinant protein was obtained per liter of bacterial culture. SDS-PAGE analysis showed a protein purity higher than 95% (Fig. 3a). Finally, western blot assays confirmed the presence of the poly-histidine epitope at the expected band size (~50 kDa) in samples in which recombinant ThSwo was induced (Fig. 3b).

Other fungal SWOs have already been produced heterologously, including those of *T. harzianum*, with yields ranging from 197.1 mg L^−1^ to 25 µg L^−1^ [13, 16, 35–38]. However, to the best of our knowledge, this is the first time that a fungal SWO has been successfully produced in its soluble and active form using an *E. coli* strain as a host. Factors such as established methodologies for genetic manipulation, low cost, and rapid growth have made *E. coli* the most widely used system for recombinant protein expression [39]. *E. coli* represents an excellent system that allows for the effective use of recombinant proteins in enzymatic biomass hydrolysis.

### Elements of ThSwo secondary structure and thermal denaturation by CD

The secondary folding of purified recombinant ThSwo was observed using circular dichroism (CD; Fig. 3c). Corroborating the modeling results (Fig. 1a, top), purified recombinant ThSwo showed a β-sheet-rich CD spectrum (Fig. 3c). Deconvolution of the ThSwo CD spectrum estimated the α-helix and β-sheet contents as 5 ± 1 and 29 ± 4%, respectively. The experimentally determined ThSwo secondary structure is in accordance with the results obtained for the native SWO of *T. reesei*, which showed α-helix and β-sheet contents of 7 and 34%, respectively, [40]. The CD analysis confirmed the efficiency of using *E. coli* to produce one fungal recombinant SWO with secondary folding resembling that of the native SWO.

The thermal stability of purified recombinant ThSwo was also analyzed using CD measurements made by monitoring the secondary structure content at 218 nm as a function of temperature, ranging from 20 to 90 °C (Fig. 3d). The heat-induced unfolding experiments showed that ThSwo has a melting temperature (*Tm*) of 48.6 ± 2 °C (Fig. 3d). The SWO from *T. reesei* shows an unusually broad optimal temperature range, from 22 to 50 °C; however, the activity decreases quite rapidly above 50 °C [16]. Our results show that the rapid decrease in SWO activity can be linked to the loss of secondary structure elements, as observed in CD unfolding experiments.

### Disruptive action of the purified recombinant ThSwo on Avicel

**Fig. 4:**
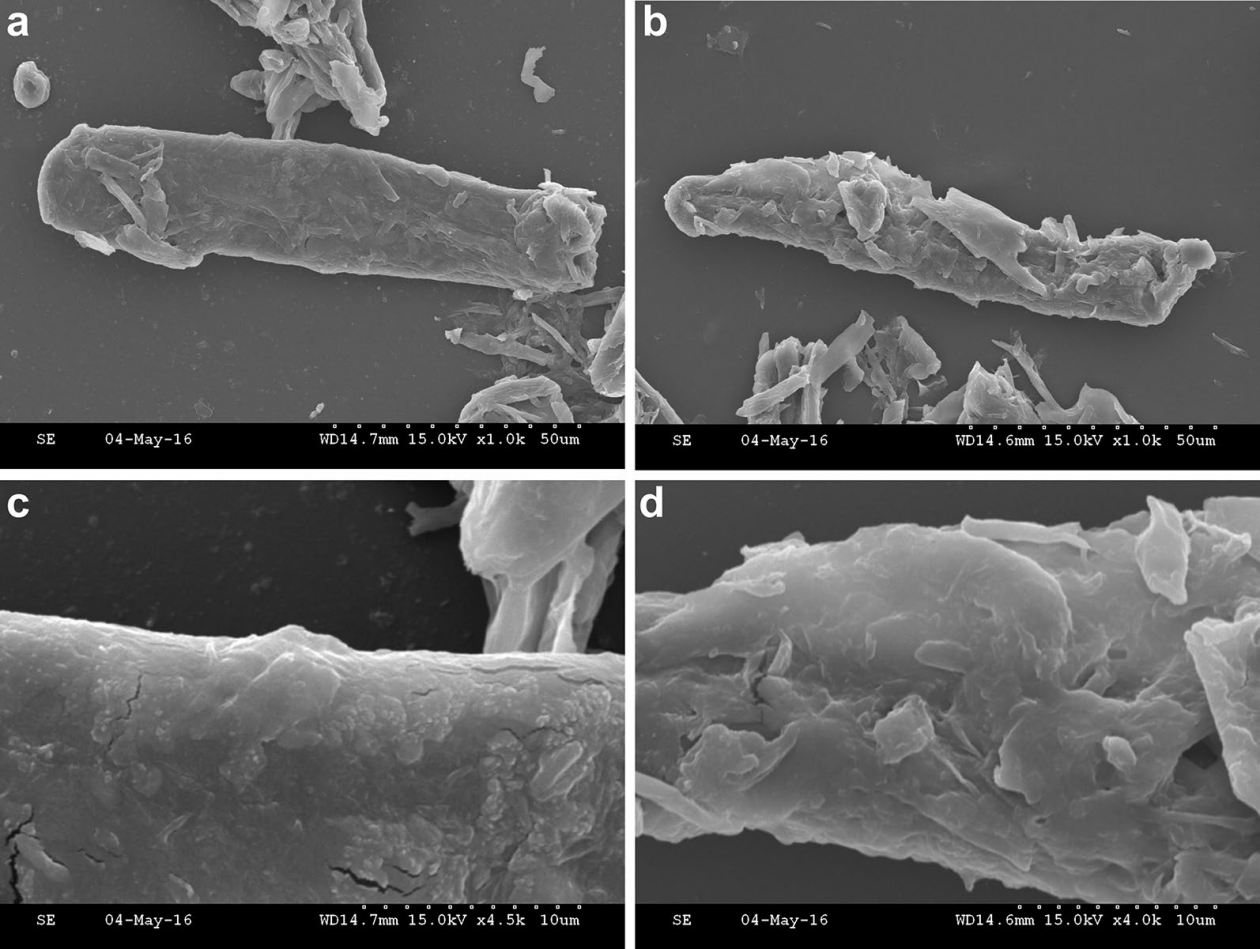
Disruptive activity of purified recombinant ThSwo on Avicel. **a**, **c** Scanning electron micrographs of Avicel in the absence or **b**, **d** presence of ThSwo. The samples were incubated in 50 mM sodium citrate buffer, pH 4.8, containing approximately 35 µg of purified recombinant ThSwo at 45 °C for 72 h. Control experiments without ThSwo or using BSA were also performed under the same conditions as mentioned above. **a**, **b**: 1000×; **c**: 4500×; and **d**: 4000×

Microscopic techniques were used to evaluate the disruptive effect of the purified recombinant ThSwo on Avicel. SWOs play an active role in the disruption of lignocellulosic structure, and SWO activity has been proposed to enhance the amorphogenesis process during biomass degradation [13, 41]. The preliminary results obtained in this study, derived via light microscopy analysis, showed a tendency toward deagglomeration of the Avicel structure in treatments that included purified recombinant ThSwo (Additional file 2: Figure S1). However, although our light microscopy findings were not conclusive, especially due to the crystalline proprieties of Avicel, the effects observed herein were similar to those reported for a previously characterized expansin-related protein [35, 42]. Meanwhile, the disruptive activity of ThSwo was confirmed by scanning electron microscopy (Fig. 4). ThSwo was able to create a rough and amorphic surface on Avicel (Fig. 4b, d). By contrast, the control samples did not show differences in the structure of Avicel, which retained a smooth surface (Fig. 4a, c).

Its disruptive activity on cellulose fibers is the major characteristic that makes SWO extremely attractive as an additive for enzymatic biomass conversion [13]. Surprisingly, our findings confirm the activity of a fungal SWO produced in a prokaryotic host that is unable to make any post-translational modifications. As previously reported, fungal SWOs are strongly glycosylated [13], and many of the purified recombinant SWOs characterized have shown an unusual migration profile on SDS-PAGE [13, 16, 36–38]. These previous findings differ from our analysis in which the protein band was detected as having a molecular mass that is consistent with the expected mass based on the amino acid sequence (Fig. 3a, b). Predictions using the GlycoEP server (http://www.imtech.res.in/raghava/glycoep/index.html) reveal that ThSwo contains at least 3 potential sites of *N*-glycosylation in addition to other predicted *O*-glycosylation sites. However, in accordance with Eibinger et al. [40], how these post-translational modifications affect SWO activity remains unknown. Future studies comparing both the recombinant ThSwo protein produced in *E. coli* and the native protein produced by *T. harzianum* may shed new light on the role of post-translational modifications on the structure and function of ThSwo.

### Synergistic effects of the purified recombinant ThSwo on xylanase catalysis

**Fig. 5:**
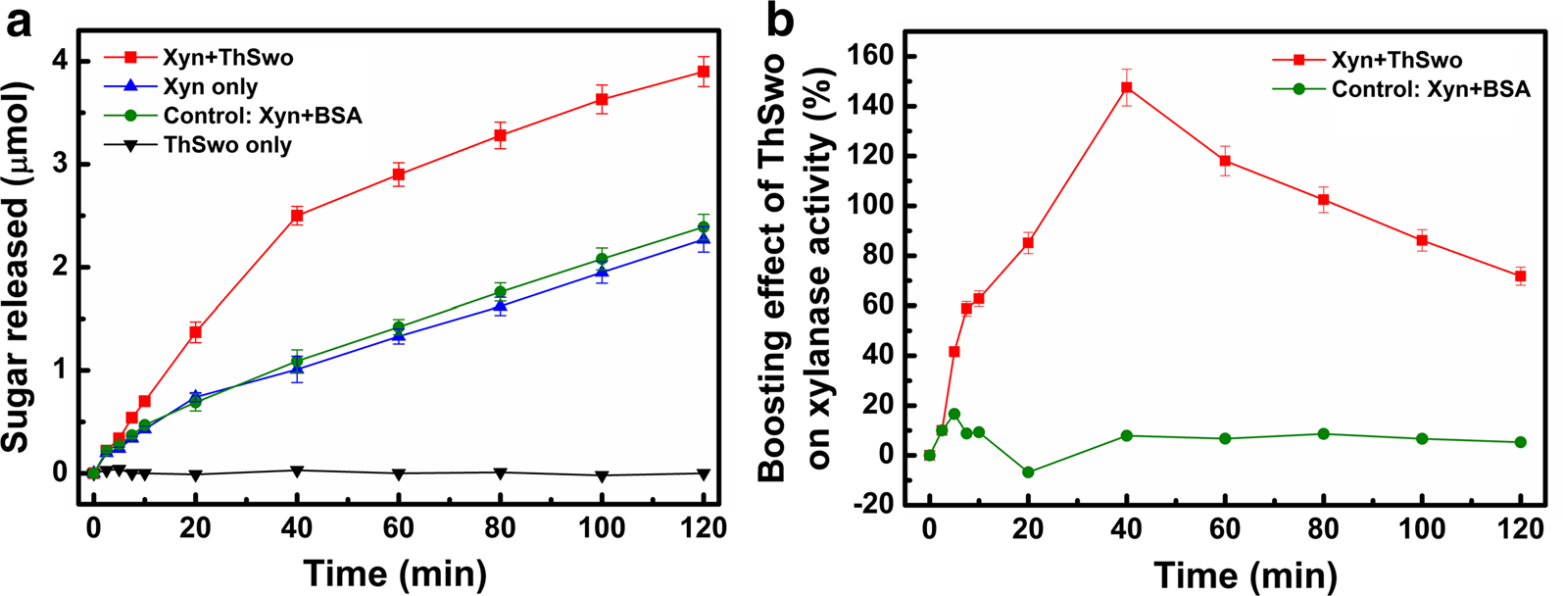
Synergistic effect of purified recombinant ThSwo on xylanase activity. **a** Time course of reducing sugars released by a commercial xylanase in the presence or absence of ThSwo. **b** Boosting effect of ThSwo on the yields of xylanase hydrolysis throughout the enzymatic reaction. Reducing sugars were quantified using the DNS method. BSA was used as a negative control. All experiments were performed in triplicate, and the *error bars* indicate the mean values ± standard deviations

The synergistic effect of the purified recombinant ThSwo was assessed by monitoring the activity of a commercial xylanase from *T. viride* on xylan samples in the presence or absence of purified recombinant ThSwo. A clear enhancement in xylanase hydrolysis performance was observed with ThSwo treatment (Fig. 5a). No differences in the yields of reducing sugars released were observed between the samples treated with BSA, used as a negative control, and those treated with xylanase alone. Similarly, treatments in which purified recombinant ThSwo was incubated alone with xylan did not result in detectable sugar reduction (Fig. 5a). Time-course results for the amounts of reducing sugars released following ThSwo treatment were used to calculate the boosting effect of ThSwo on xylanase activity (Fig. 5b). A range of boosting effects caused by ThSwo on xylanase activity was observed throughout the enzymatic reaction, with the highest value of 147 ± 7% being observed 40 min after the reaction was initiated (Fig. 5b).

The synergistic effect of expansin-related proteins to improve the enzymatic degradation of complex cellulosic substrates, including sugarcane bagasse, corn stover, switchgrass, chitin, filter paper, and xylan, have been reported previously [10, 15, 20, 36, 42–44], with the overall improvements in enzymatic activity reaching 364% [44]. Our findings showed that purified recombinant ThSwo was able to improve the xylanase activity up to 147 ± 7%; similar results were also obtained with the recombinant ThSwo produced in *Aspergillus niger*, which was able to increase the hydrolytic efficiency of a commercial cellulase cocktail by twofold (protein production data was not presented) [20]. Interestingly, our results showed that the effects of ThSwo on enhancing xylanase performance by ThSwo were prominent, especially at the beginning of the enzymatic reaction. Corroborating the results of Gourlay et al. [43], ThSwo alone was not able to directly and efficiently hydrolyze pure xylan and did not result in detectable sugar reduction, suggesting that fungal SWO acts primarily by promoting amorphogenesis within the ordered xylan structure, explaining these effects and indicating that xylanases are excellent models to evaluate their synergistic effects [43]. Furthermore, evidence showing that both *T. harzianum* and *T. reesei* co-induce the *swo* and *xyn* genes [31, 32], which are closely positioned in these fungi genomes, strongly suggests that the synergistic effect between SWOs and xylanases must be fine-tuned in vivo.

## Conclusions

New alternatives to improve biomass conversion and to overcome the physical and chemical barriers that exist in lignocellulosic substrates are needed, representing one of the major challenges facing biorefineries. In this context, fungal SWOs are promising additives for use in enzyme cocktails to improve the overall performance of biomass hydrolysis. However, many aspects of the structure and mechanism of action of fungal SWOs remain unknown. In the present study, we were able to produce a recombinant, active SWO from *T. harzianum* using *E. coli* as a host and showed its potential synergistic effect on xylanase activity. Thus, our work paves the way for further studies evaluating the structure and function of this protein, especially with regard to its biotechnological use.

## Methods

### Strains, media and chemicals

The *T. harzianum* IOC-3844 strain was provided by the Oswaldo Cruz Institute (Rio de Janeiro, RJ, Brazil) and used for RNA extraction, cDNA synthesis and gene cloning. The *E. coli* strains DH10B (Thermo Fisher Scientific, Waltham, MA, USA) and Rosetta-gami 2 (DE3) (Novagen, Madison, WI, USA) were used as the host strains for cloning and heterologous protein expression, respectively. The oligonucleotides primers were synthesized by Exxtend, Ltd. (Campinas, SP, Brazil). The pET28a(+) vector, used for protein expression, was obtained from Novagen. The *Nde*I and *Xho*I restriction endonucleases were purchased from New England Biolabs (Ipswich, MA, USA). The protease inhibitor phenylmethanesulfonyl fluoride (PMSF), tris(2-caroxy-thyl)phosphine (TCEP), lysozyme, commercial xylanase from *Trichoderma viride* and Avicel^®^ were purchased from Sigma-Aldrich (St. Louis, Missouri, USA). All other chemicals used were of biochemical research grade.

### ThSwo domain identification, protein modeling and phylogenetic analysis

ThSwo protein domain identification was conducted using the simple modular architecture research tool (SMART; http://smart.embl-heidelberg.de/) [25]. Protein structure and function prediction was made using the I-TASSER server (http://zhanglab.ccmb.med.umich.edu/I-TASSER/) [45]. The best-ranked N- and C-termini models generated by the I-TASSER server were used for structural protein alignment. ThSwo N- and C-termini models were superimposed on the C-terminal domain of cellobiohydrolase I from *T. reesei* (PDB code: 2CBH; [28]) and the structure of EXPB1, a beta-expansin and group-1 pollen allergen from maize (PDB code: 2HCZ; [29]), respectively, using PYMOL software (Schrodinger LLC, New York, NY, USA).

The phylogenetic relationships among ThSwo, plant expansins, expansin-related proteins from bacteria, fungi, amoebozoa and nematodes, pollen allergen proteins and family-45 glycosyl hydrolases (GH45) were analyzed using MEGA6 software [46]. Initially, the protein sequences that showed the best hits in the BLAST analyses using the ThSwo sequence as a query were selected in the UniProt database (http://www.uniprot.org/) (Additional file 1: Table S1). The sequences were aligned with ClustalW [47] as implemented using MEGA6 software with a 10/3 gap opening penalty and a 0.1/5 gap extension penalty between pairwise alignment and multiple alignment, respectively. The phylogenetic tree was estimated using the Neighbor-Joining method [48], and the aligned sequences were bootstrapped 1000 times for each analysis [49]. Pairwise deletion was employed to address alignment gaps and missing data. FigTree software (http://tree.bio.ed.ac.uk/software/figtree/) was used for tree visualization and final figure editing.

### RNA-Seq data, proteomics approaches and ThSwo production in *E. coli*

RNA-Seq data for *T. harzianum*, obtained during its plant biomass-induced growth [33], were used to quantify ThSwo gene expression under these conditions, as described by Santos et al. [34]. Subsequently, the transcriptional results for ThSwo were compared with available data from the direct detection of ThSwo in the *T. harzianum* secretome [20] under the same culture conditions.

The coding sequence of the ThSwo protein (GenBank: KM555252.1) lacking the signal peptide sequence (amino acid residues 1–25) was amplified using PCR with cDNA from the *T. hazianum* IOC-3844 strain using the specific oligonucleotides ThSwoF (5′-AGTATCATATGGGCCAATGTGG-3′) and ThSwoR (5′-CAGAGCTCGAGTTAGTTTTGACTAA-3′), which contain restriction sites for the enzymes *Nde*I and *Xho*I, respectively. Then, the PCR product was cloned into a pET28a(+) expression vector (Novagen) to enable expression as a fusion protein with an N-terminal tag containing six histidine residues (His_6_-tag). Nucleotide base substitutions in the recombinant plasmid were analyzed through DNA sequencing.

ThSwo was overexpressed in *E. coli* Rosetta-gami 2 (DE3) cells (Novagen, Madison, WI, USA). The cells were cultured at 37 °C with shaking at 250 rpm in 1 L of LB broth containing 0.2% (w/v) glucose, chloramphenicol (34 µg mL^−1^) and kanamycin (30 µg mL^−1^) until an OD_600_ of 1.0 was reached. Recombinant protein expression was induced by adding 0.4 mM isopropyl-β-d-thiogalactopyranoside (IPTG), followed by cultivation for 16 h at 16 °C and 160 rpm. The culture was then centrifuged (3000*g*, 15 min, 4 °C), and the cells were resuspended in 25 mL of buffer A [40 mM HEPES pH 7.5, 150 mM NaCl, 10 mM 2-Mercaptoethanol and 10% (v/v) glycerol] containing 1 mg mL^−1^ lysozyme, 1 mM PMSF, and 0.1% (v/v) Tween 20. The cells were disrupted using sonication, and the soluble fraction was collected by centrifugation (20,000*g*, 40 min, 4 °C). The purification of ThSwo was achieved through two chromatographic steps (nickel affinity and gel filtration) using columns (prepacked Ni Sepharose High Performance HisTrap and HiPrep 16/60 Sephacryl S-100 HR columns) previously equilibrated with buffer A, coupled to an ÄKTA FPLC device (GE Life Sciences). The concentrations of the purified proteins were determined spectroscopically using the molar extinction coefficient (ε_280_) predicted based on the ThSwo amino acid sequence. Purity of the ThSwo protein was estimated with 12% SDS-PAGE staining with Coomassie brilliant blue R-250. For western blot analysis, the proteins from SDS-PAGE were blotted onto nitrocellulose membranes (ImmobilonTM-Nc; Sigma) using a Trans-Blot Semi-Dry Transfer Cell (Bio-Rad, Hercules, CA, USA) in accordance with the manufacturer’s instructions. Recombinant ThSwo was detected with a 1:4000 dilution of a His-probe antibody and a 1:8000 dilution of alkaline phosphatase-conjugated anti-rabbit IgG, both provided by Santa Cruz Biotechnology Inc. (Santa Cruz, CA, USA).

### Secondary folding and thermal-induced unfolding by circular dichroism

The far-UV CD spectra of ThSwo were collected using a Jasco model J-815 CD spectropolarimeter (Japan Spectroscopic; Tokyo, Japan) coupled to a Peltier control system (PFD 425S-Jasco). The CD spectra were generated using the purified recombinant ThSwo protein at a concentration of approximately 4 µM in buffer A containing 1 mM of TCEP at 20 °C. A total of 12 accumulations within the range of 260–208 nm at a rate of 50 nm min^−1^ were recorded and averaged using a quartz cuvette with a pathlength of 1 mm. The Dichroweb server (http://dichroweb.cryst.bbk.ac.uk/html/home.shtml) and CDNN deconvolution software were used to statistically analyze the CD spectra [50]. The ThSwo thermal-induced unfolding experiments followed by CD were measured at 218 nm from 20 to 90 °C, using approximately 10 µM ThSwo in buffer A containing 1 mM of TCEP with a 1 mm pathlength cell. The melting temperature (*Tm*) of ThSwo was obtained by fitting the CD data to a sigmoidal Boltzmann function as implemented using Origin 8.1 software (OriginLab, Northampton, MA, USA).

### Light and scanning electron microscopic analyses

One milligram of Avicel PH-101 (Sigma-Aldrich) was incubated in 50 mM sodium citrate buffer, pH 4.8, containing approximately 35 µg of purified recombinant ThSwo at 45 °C for 72 h. Control experiments without ThSwo or using BSA were also performed under the same conditions as mentioned above. The effect of ThSwo on Avicel fibers was initially observed using light microscopy with a B-383FL microscope (Optika Microscopes, Ponteranica, Italy). Afterwards, the samples were rinsed with water and dried to completely remove all residual solvent, and they were then used for scanning electron microscopy. Photomicrographs of the samples were taken using a scanning electron microscope (Hitachi S-2600 N, Tokyo, Japan) at a voltage of 15 kV. A total of three replicates were performed for each experiment.

### Synergistic effect of ThSwo on xylanase activity

To determine if the purified recombinant ThSwo can increase the enzymatic activity of a commercial xylanase, samples of 10 mg mL^−1^ xylan in 50 mM sodium citrate buffer pH 4.8, in the presence or absence of 100 µg of purified recombinant ThSwo or 100 µg BSA were preincubated at 45 °C for 18 h with gentle agitation. Following incubation, a commercial xylanase from *T. viride* (0.25 U; Sigma-Aldrich) was added in a 1 mL reaction and incubated for 120 min at 45 °C. At different reaction time intervals, 100 µL of the reaction supernatant were collected, centrifuged at 16,000 rpm for 5 min at room temperature, mixed with the same volume of 3,5-dinitrosalicylic acid (DNS) and boiled for 5 min. The amount of reducing sugars released was determined using the DNS method [51]. The boosting effect of the purified recombinant ThSwo on xylanase activity was calculated as follows:

$$\text{ThSwo} \, \text{boosting} \, \text{effect} \,( \%) = \left[ {\frac{{x^{\prime}}}{x} - \text{1}} \right] \times 100$$

where $x$ is the amount of reducing sugars produced by xylanase alone, and $x^{\prime}$ is the amount of reducing sugars produced by xylanase in the presence of the purified recombinant ThSwo.

## Additional files

**Additional file 1: Table S1.** Expansin-related proteins from bacteria, fungi, amoebozoa, nematodes and plants used for the ThSwo phylogenetic analysis.

**Additional file 2: Figure S2.** Light microscopy (10×) of Avicel in the presence or absence of purified recombinant ThSwo for 72 h at 45 °C. **a** Avicel in the absence of ThSwo at 0 h. **b** Avicel in the absence of ThSwo at 72 h. **c** Avicel in the presence of ThSwo at 48 h. **d** Avicel in the presence of ThSwo at 72 h. As an additional control, Avicel was also treated with BSA under the same conditions and observed.

## Abbreviations

SWOs: swollenins
ThSwo: swollenin from *T. harzianum*
fCBD: fungal-type cellulose-binding domain
DPBB: double-psi beta-barrel fold
GH45: family-45 glycosyl hydrolase
PDB: protein data bank
CD: circular dichroism
