# Supplementary material for: Production of a recombinant swollenin from Trichoderma harzianum in Escherichia coli and its potential synergistic role in biomass degradation
Source: Microb Cell Fact. 2017 May 16;16:83. doi: 10.1186/s12934-017-0697-6 (PMC5432999; doi:10.1186/s12934-017-0697-6)
Supplement: Supplementary file 1 — Additional file 1: Table S1. Expansin-related proteins from bacteria, fungi, amoebozoa, nematodes and plants used for the ThSwo phylogenetic analysis. [file 12934_2017_697_MOESM1_ESM.pdf]

Table S1. Expansin-related proteins from bacteria, fungi, amoebozoa, nematodes and plants used for the ThSwo phylogenetic analysis.

Name	Organism	Protein	Size (aa)	UnprotKb ID	GO-Biological process*
Swollenin/ Swollenin, putative					
Th-IOC3844	*Trichoderma harzianum*	Swollenin	499	-	Carbohydrate metabolic process
Nf-Q4WD28	*Neosartorya fumigata*	Swollenin, putative	500	Q4WD28	Carbohydrate metabolic process
Tr-Q9P8D0	*Trichoderma reesei*	Swollenin	493	Q9P8D0	Carbohydrate metabolic process
Ta-B3FRA5	*Trichoderma asperellum*	Swollenin	495	B3FRA5	Carbohydrate metabolic process
Tp-A8D452	*Trichoderma pseudokoningii*	Swollenin	494	A8D452	Carbohydrate metabolic process
Pd-B5M079	*Penicillium decumbens*	Swollenin	499	B5M079	Carbohydrate metabolic process
Ts-B8M618	*Talaromyces stipitatus*	Swollenin, putative	483	B8M618	Carbohydrate metabolic process
Nf-B0YDB9	*Neosartorya fumigata*	Swollenin, putative	478	B0YDB9	Carbohydrate metabolic process
Tr-A0A024RZP7	*Trichoderma reesei*	Swollenin	493	A0A024RZP7	Carbohydrate metabolic process
Po-S7ZAB6	*Penicillium oxalicum*	Swollenin, putative	499	S7ZAB6	Carbohydrate metabolic process
Po-F2X0X8	*Penicillium oxalicum*	Swollenin	499	F2X0X8	Carbohydrate metabolic process
Ac-Q207U8	*Acanthamoeba castellanii*	Swollenin/expansin-like protein	144	Q207U8	Plant-type cell wall organization
Af-A0A0J5PHY3	*Aspergillus fumigatus*	Swollenin, putative	438	A0A0J5PHY3	-
Tc-A0A0A8IBA8	*Talaromyces cellulolyticus*	Uncharacterized protein	491	A0A0A8IBA8	Carbohydrate metabolic process
Tm-B6Q799	*Talaromyces marneffei*	Swollenin, putative	374	B6Q799	-
Tc-A0A0B8N741	*Talaromyces cellulolyticus*	Swollenin	389	A0A0B8N741	-
Eh-M7WPU7	*Entamoeba histolytica*	Swollenin/expansin family protein	442	M7WPU7	-
Eh-N9V1P0	*Entamoeba histolytica*	Swollenin/ expansin family protein, putative	442	N9V1P0	-
Eh-M2RHW5	*Entamoeba histolytica*	Swollenin/ expansin family protein	442	M2RHW5	-
Expansin					
Os-Q40638	*Oryza sativa*	Expansin-B1	267	Q40638	Cell wall organization, sexual reproduction
At-Q9LDJ3	*Arabidopsis thaliana*	Expansin-A12	252	Q9LDJ3	Plant-type cell wall organization

Zm-P58738	*Zea mays*	Expansin-B1	269	P58738	Cell wall organization, sexual reproduction
Os-Q40636	*Oryza sativa*	Expansin-A2	251	Q40636	Plant-type cell wall organization
Os-A2Y5R6	*Oryza sativa*	Expansin-A4	246	A2Y5R6	Plant-type cell wall organization
Os-Q8H7T4	*Oryza sativa*	Expansin-B10	267	Q8H7T4	Cell wall organization, sexual reproduction
Bs-D3JXR9	*Bacillus subtilis*	Expansin-YoaJ	232	D3JXR9	-
Sa-A0A0L0KKF5	*Streptomyces acidiscabies*	Uncharacterized protein	256	A0A0L0KKF5	Plant-type cell wall organization
Ku-W7SH78	*Kutzneria* sp.	Expansin-A15	250	W7SH78	Plant-type cell wall organization
Bl-A0A0W8KAT0	*Bacillus licheniformis*	Expansin-yoaJ	232	A0A0W8KAT0	-
Pb-A0A172ZHD6	*Paenibacillus bovis*	Expansin-YoaJ	236	A0A172ZHD6	-
Gp-G9FYT3	*Globodera pallida*	Expansin B3	256	G9FYT3	Carbohydrate metabolic process
Gr-C8AWF8	*Globodera rostochiensis*	Expansin B2	154	C8AWF8	-
Ha-J7JLW4	*Heterodera avenae*	Expansin	285	J7JLW4	Carbohydrate metabolic process
Dd-F1ADL3	*Ditylenchus destructor*	Expasin-like protein	300	F1ADL3	Carbohydrate metabolic process
Bx-X2C1J4	*Bursaphelenchus xylophilus*	Expasin-like protein		X2C1J4	-
Pollen allergen					
Ag-P38948	*Alnus glutinosa*	Major pollen allergen Aln g 1	160	P38948	Plant defense
Dc-O04298	*Daucus carota*	Major allergen Dau c 1	154	O04298	Defense response, response to biotic stimulus
Qa-B6RQS2	*Quercus alba*	Pollen allergen Que a 1 isoform	160	B6RQS2	Defense response, response to biotic stimulus
Cb-B6RQR8	*Carpinus betulus*	Pollen allergen Car b 1 isoform	160	B6RQR8	Defense response, response to biotic stimulus
Family-45 Glycosyl hydrolase					
Th-A0A0F9XY55	*Trichoderma harzianum*	PHB depolymerase family esterase	250	A0A0F9XY55	Carbohydrate metabolic process
Tr-P43317	*Trichoderma reesei*	Endoglucanase-5	242	P43317	Cellulose catabolic process
Tv-Q7Z7X0	*Trichoderma viride*	Endoglucanase V	247	Q7Z7X0	Carbohydrate metabolic process

Nc-Q1K5M0	*Neurospora crassa*	Endoglucanase V	293	Q1K5M0	Carbohydrate metabolic process
Ro-Q8J1L1	*Rhizopus oryzae*	Endo-glucanase RCE2	360	Q8J1L1	Carbohydrate metabolic process
Sc-B5BNY1	*Staphylotrichum coccosporum*	Endo-beta-D-1,4-glucanase	316	B5BNY1	Carbohydrate metabolic process
Pd-B5AKD1	*Penicillium decumbens*	GH45 protein	266	B5AKD1	Carbohydrate metabolic process
Gz-Q69F59	*Gibberella zeae*	GH45 protein	355	Q69F59	Carbohydrate metabolic process
Cj-P18126	*Cellvibrio japonicus*	Endoglucanase B	511	P18126	Cellulose catabolic process
Tt-C5BPF5	*Teredinibacter turnerae*	Endoglucanase	390	C5BPF5	Carbohydrate metabolic process

*Data from UniProt (http://www.uniprot.org/)
