# Supplementary material for: Production of a recombinant swollenin from Trichoderma harzianum in Escherichia coli and its potential synergistic role in biomass degradation
Source: Microb Cell Fact. 2017 May 16;16:83. doi: 10.1186/s12934-017-0697-6 (PMC5432999; doi:10.1186/s12934-017-0697-6)
Supplement: Supplementary file 2 — Additional file 2: Figure S2. Light microscopy (10×) of Avicel in the presence or absence of purified recombinant ThSwo for 72 h at 45 °C. a Avicel in the absence of ThSwo at 0 h. b Avicel in the absence of ThSwo at 72 h. c Avicel in the presence of ThSwo at 48 h. d Avicel in the presence of ThSwo at 72 h. As an additional control, Avicel was also treated with BSA under the same conditions and observed. [file 12934_2017_697_MOESM2_ESM.pdf]

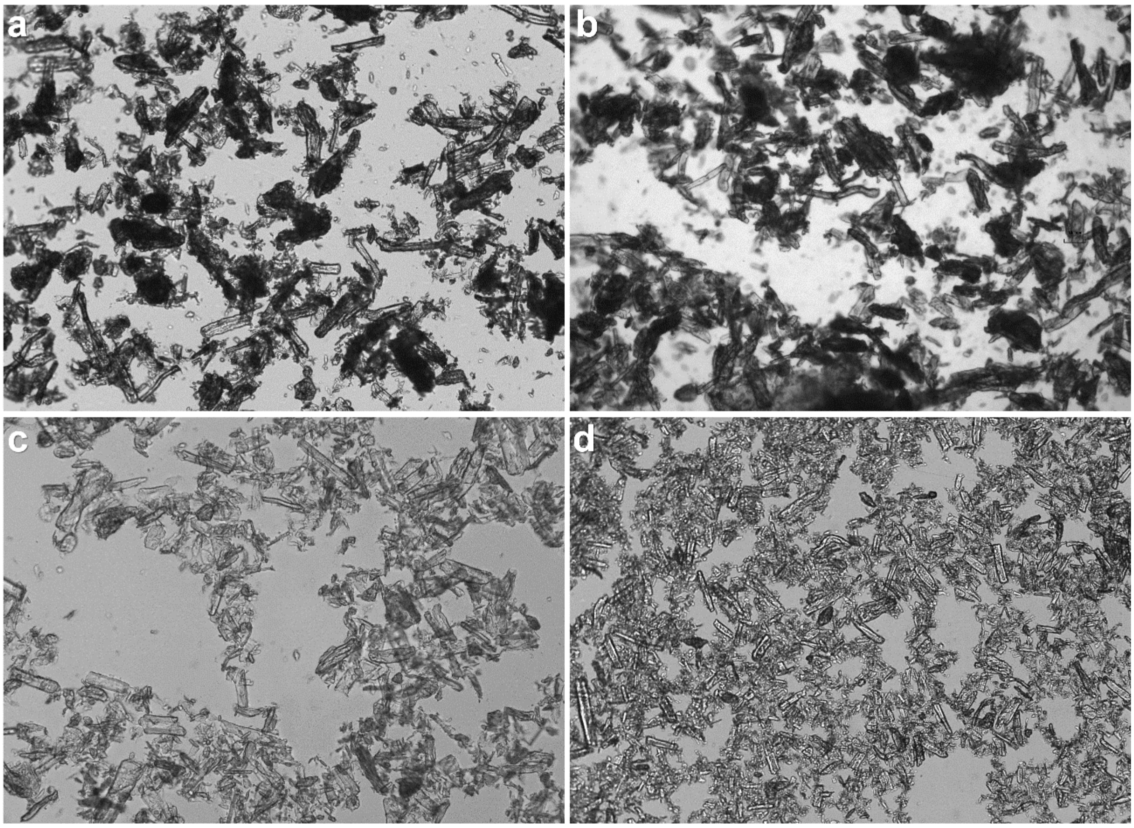

Additional file 2: Fig. S1. Light microscopy (10x) of Avicel in the presence or absence of purified recombinant ThSwo for 72 h at 45°C. A) Avicel in the absence of ThSwo at 0 h. B) Avicel in the absence of ThSwo at 72 h. C) Avicel in the presence of ThSwo at 48 h. D) Avicel in the presence of ThSwo at 72 h. As an additional control, Avicel was also treated with BSA under the same conditions and observed.
